# Improvement of catalytic performance of lignin peroxidase for the enhanced degradation of lignocellulose biomass based on the imbedded electron-relay in long-range electron transfer route

**DOI:** 10.1186/s13068-016-0664-1

**Published:** 2016-11-15

**Authors:** Le Thanh Mai Pham, Su Jin Kim, Yong Hwan Kim

**Affiliations:** 1 School of Energy and Chemical Engineering, UNIST, 50 UNIST-gil, Ulju-gun, Ulsan, 44919 Republic of Korea; 2 Life Ingredient Material Research Institute, CJ Company, 42 Gwanggyo-ro, Yeongtong-gu, Suwon-si, Gyeonggi-do Republic of Korea

**Keywords:** *Phanerochaete chrysosporium*, Lignin peroxidase isozyme H8, Radical coupling, Suicide inhibition

## Abstract

**Background:**

Although lignin peroxidase is claimed as a key enzyme in enzyme-catalyzed lignin degradation, in vitro enzymatic degradation of lignin was not easily observed in lab-scale experiments. It implies that other factors may hinder the enzymatic degradation of lignin. Irreversible interaction between phenolic compound and lignin peroxidase was hypothesized when active enzyme could not be recovered after the reaction with degradation product (guaiacol) of lignin phenolic dimer.

**Results:**

In the study of lignin peroxidase isozyme H8 from white-rot fungi *Phanerochaete chrysosporium* (LiPH8), W251 site was revealed to make the covalent coupling with one moiety of monolignolic radical (guaiacol radical) by LC-MS/MS analysis. Hypothetical electron-relay containing W251 residue was newly suggested based on the observation of repressed radical coupling and remarkably lower electron transfer rate for W215A mutant. Furthermore, the retardation of the suicidal radical coupling between the W251 residue and the monolignolic radical was attempted by supplementing the acidic microenvironment around the W251 residue to engineer radical-robust LiPH8. Among many mutants, mutant A242D showed exceptional catalytic performances by yielding 21.1- and 4.9-fold higher increases of k_cat_ and k_cat_/K_M_ values, respectively, in the oxidation of non-phenolic model lignin dimer.

**Conclusions:**

A mechanism-based suicide inhibition of LiPH8 by phenolic compounds was firstly revealed and investigated in this work. Radical-robust LiPH8 was also successfully engineered by manipulating the transient radical state of radical-susceptible electron-relay. Radical-robust LiPH8 will play an essential role in degradation of lignin, which will be consequently linked with improved production of sugars from lignocellulose biomass.

**Electronic supplementary material:**

The online version of this article (doi:10.1186/s13068-016-0664-1) contains supplementary material, which is available to authorized users.

## Background

Lignin is the natural substrate of ligninolytic peroxidase, even though it is bulky and very recalcitrant toward degradation. Lignin peroxidase (LiP) and versatile peroxidase (VP) have been demonstrated to directly oxidize a non-phenolic lignin model compound (veratrylglycerol-beta-guaiacyl ether, VE dimer) [1, 2]. It is quite unfeasible for this large model compound and lignin polymer to gain access to heme through a channel, whose channel opening to heme is even smaller than in classical plant peroxidases. Lignin peroxidases from white-rot fungi, lignin peroxidase isozyme H8 (LiPH8) from *Phanerochaete chrysosporium* harbors exposed catalytic W171 site which was demonstrated to play a vital role in the oxidation of high-redox potential substrates such as veratryl alcohol (VA) or non-phenolic lignin derivatives. The oxidation was manipulated through a long-range electron transfer (LRET) to the heme (for both compound I and compound II intermediates) [3]. The distinct roles of the surface-active site in the oxidation of high-redox potential substrates or bulky lignin macromolecules were also investigated for VP from *Pleurotus eryngii*. This property allows VP to oxidize synthetic model dimers [2] and water-soluble sulfonated lignins [4].

In nature, efficient lignin degraders, white-rot fungi, secrete enzymes collectively termed “ligninases” in which the most important and active enzyme is lignin peroxidase. However, in vitro enzymatic degradation of lignin has not been easily observed in lab-scale experiments, and it implies that other factors may hinder the enzymatic degradation of lignin.

The properties of thermostability and the tolerance at acidic pH values of VP from *P. eryngii* were reported to be improved through studies of an ancestral mutation method or comparative structural analysis [5, 6]. Besides those limitations, the inhibitor interaction between the enzyme and the phenolic compound was emphasized as a significant factor which disrupts LRET and catalytic turnover of non-phenolic lignin dimer [7].

In this study, the enzyme mechanism-based inhibition mode of the phenolic compound was investigated. The site responsible for the irreversible interaction between LiPH8 and free hydroxyl monolignol was searched by LC-MS/MS analysis. Surprisingly, the W251 site was identified as a suicide site by coupling with the guaiacol radical (the product released from the degradation of VE dimer) and proved to be an essential electron-relay residue on the LRET route from the surface-active site W171 to heme. Its role as a stepping stone in the hopping ET mechanism was demonstrated through the rational mutagenesis of its aromatic character. Creating an acidic environment around the radical coupling site to prevent coupling with the phenoxy radical was also examined for the rational design of effective LiP. With this purpose, a combination of liquid chromatography-tandem mass spectrometry, stopped-flow spectrophotometry, and rational mutagenesis techniques was used. As far as we know, this is the first successful trial to increase the catalytic performance of LiPH8 by altering the intramolecular ET route from the surface site to heme.

## Methods

### Materials

Hydrogen peroxide, hemin, oxidized glutathione, ampicillin, isopropyl-b-d-thiogalactopyranoside, 2,2-azino-bis (3-ethylbenzothiazoline-6-sulfonate) (ABTS), guanidine hydrochloride, dibasic potassium phosphate, citric acid, trizma hydrochloride, and guaiacol used in this study were purchased from the Sigma Chemical Co., South Korea and were used without any further purification. Veratrylglycerol-beta-guaiacyl ether (VE dimer) at 97% purity was obtained from AstaTech Inc., USA.

### Recombinant enzyme preparation

The LiPH8 synthetic gene, including the seven-residue pro-sequence, was synthesized by the Bioneer Company (South Korea). The gene coding protein sequence was retrieved from a previously published report [8] (UniProtKB entry: P06181). The refolding and purification procedures were performed as previously reported [8].

The mutant LiPH8 genes were constructed using a one-step PCR method [9]. The procedure involves a one-step PCR reaction using plasmid pET-LiPH8 as a template and synthesized oligonucleotide primers containing the desired mutations, with each complementary to the opposite strands of the vector.

### Liquid chromatography-tandem mass spectrometry (LC-MS/MS) analysis of modified lignin peroxidase

The purified LiPH8 enzyme (15 μM) which was prepared in 0.1 M tartrate buffer pH 4.0 reacted with guaiacol (100 μM) in the presence of 100 μM H_2_O_2_ as the final concentration (inactivated sample). The control sample was prepared under similar conditions in the absence of H_2_O_2_. After 1 h of reaction time, the protein samples (approximately 5 μg/lane) were separated on a 12% polyacrylamide gel and subsequently stained with colloidal Coomassie Brilliant Blue G-250 (CBB). The stained protein bands were excised and subjected to tryptic digestion as previously described [10]. Sample purification and preparation techniques were based on nano-scale reversed-phase columns for the sensitive analysis of complex peptide mixtures by matrix-assisted laser desorption/ionization mass spectrometry.

Nano LC-MS/MS analysis was performed with a nano-HPLC system (Agilent, Wilmington, DE, USA). The nano-chip column (Agilent, Wilmington, DE, USA, 150 mm × 0.075 mm) was used for peptide separation.

Mobile phase A for the LC separation was 0.1% formic acid in deionized water, and mobile phase B was 0.1% formic acid in acetonitrile. The chromatography gradient was designed for a linear increase from 3% B to 50% B in 25 min, 90% B in 5 min, and 3% B in 15 min. The flow rate was maintained at 300 nL min^**−1**^.

Product ion spectra were collected in the information-dependent acquisition (IDA) mode and were analyzed by an Agilent 6530 Accurate-Mass Q-TOF using continuous cycles of one full TOF MS scan from 350 to 1200 *m/z* (1.0 s) plus two product ion scans from 100 to 1700 *m/z* (1 s each). Precursor *m/z* values were selected starting with the most intense ion using a selection isolation width of approximately 4 Da. The rolling collision energy feature was used, which determines the collision energy based on the precursor value and charge state. The dynamic exclusion time for precursor ion *m/z* values was 20 s.

The Mascot algorithm (Matrix Science Ltd, UK) was used to identify peptide sequences present in a protein sequence database. The MS tolerance was 100 ppm, and the MS/MS tolerance was 0.1 Da. Peptides resulting from tryptic digestion were only considered for data analysis.

### Steady-state reactions

In order to obtain kinetic parameters, the oxidation reaction was performed with the VE dimer. Kinetic investigations of the VE dimer were conducted at concentrations ranging from 50 to 2000 µM VE dimer in the presence of 0.015 µM enzyme. The reaction was initiated by the addition of H_2_O_2_ at a fixed concentration of 250 µM at 25 °C. The absorbance at 310 nm was recorded by a spectrophotometer within 30 s of oxidation and was correlated to the amount of veratraldehyde (VAD) formed as a degradation product using an extinction coefficient of 9.3 mM^−1^ cm^−1^.

The net oxidation rate was evaluated by examining the amount of consumed substrate in the presence of enzyme and H_2_O_2_ after subtracting the value measured in the presence of H_2_O_2_ alone. All of the data reported are the mean of triplicate experiments. Steady-state kinetic parameters were obtained from the rearrangement of the Hanes–Woolf plot from the Michaelis–Menten equation.

### Transient kinetic reactions

The kinetic studies of compound I formation and decay were performed with an SX20 stopped-flow device (Applied Photophysics Co., UK) equipped with a Monochromator rapid-scanning diode array detector (Applied Photophysics Co., UK). First-order rate constants of compound I decay (*k*_obs-1_) were calculated from the absorbance changes at 417 nm (isosbestic point of compound II and the resting state) [11].

### H_2_O_2_-dependent oxidation of VE dimer

Oxidation of VE dimer (2000 µM) was catalyzed by LiPH8 (0.075 µM) in the presence of H_2_O_2_ in the range of 50–1000 µM. The oxidation reaction was performed in 0.1 M sodium tartrate buffer pH 4.0 at 25 °C. After 4 h, the reaction was subjected to HPLC analysis for detection of VAD as released product. The HPLC analysis procedure was performed as previously reported work [7]. HPLC analysis was performed using an Agilent 1200 HPLC system with samples injected onto a reverse-phase Eclipse XDB-C18 column (4.6 × 150 mm, 5 µm, Agilent). Stepwise gradient separation, from 0.1% aqueous trifluoroacetic acid (solvent A) to methanol–acetonitrile (25:75; v/v; solvent B), was performed under the following conditions: flow rate = 1.5 mL/min; column temperature = 30 °C; and 15% B at 0 min, 30% B at 2 min, 60% B at 11 min, 100% B at 11.5 min, and 0% B at 13 min. The experiment was performed in duplicate. Oxidation efficiency was evaluated through the ratio values of the supplied H_2_O_2_ and the formed VAD concentration for WT and mutants.

### pH dependence of steady-state kinetic parameters

The pH-dependent oxidation of VE dimer was measured as described above. Citric acid–sodium hydrogen phosphate buffer solutions were used for varying the pH in the range of 2.6–3.8. Enzyme LiPH8 was incubated in the reaction buffer in the presence of VE dimer for 5 min before H_2_O_2_ was added to start the oxidation reaction.

### Modeling of the mutated structure and pK_a_ prediction

The protein structure was achieved as PDB ID: 1B82 and submitted to the RosettaBackrub server for a point mutation to generate modeled structures of mutated variants [12]. The number of generated structures was set to 20. The radius, which is subject to backrub flexible backbone modeling, was set to be within 6 Å around the target site.

A hydrogen atom was added to the structure by the Mobility server [13]. Structural models for up to 10 of the best-scoring structures were subjected to the PDB2PQR server to predict the pK_a_ values of ionizable groups in the protein [14, 15].

All the protein molecular structures in this study were visualized using the program Molegro Molecular Viewer (MMV 2.5.0; http://www.clcbio.com/products/molegro/#molecular-viewer). The 2D chemical structure and reaction scheme were drawn by using program ChemDraw 8.0.

### Density functional theory (DFT) calculations for proposed redox centers in LiPH8

Single-point calculations were carried out with the Gaussian 03 program. The hybrid B3LYP functional and 6–311G** basis set were used for structural optimization. All of the species (resting state and cationic radical) were structurally optimized in the gas phase, and frequency calculations were performed for the optimized structures. The energies were recomputed by single-point calculations of the optimized structures by the 3–21G^+^** basis set. Calculated data of the H176/Heme redox center were retrieved from a previously reported study [16].

## Results

### Identification of the radical coupling site of LiPH8

In a previously reported study, the inactivation was only observed in catalysis of high-redox potential substrates (VA and VE dimer) which were oxidized by surface-active site W171 through LRET pathway. One of the most active inhibitors is guaiacol which is detected as a product of the degradation of the model lignin dimer [7]. The irreversible interaction between inhibitor and enzyme was suggested when recovery of the enzyme could not be obtained after reacting with free hydroxyl phenolics (data not shown). This suggested that the irreversible modification may take place during the catalysis cycle which led to formation path of inactive enzyme form rather than closing the catalysis cycle.

Purified enzymes were prepared which Rz values (A_409_/A_280_) were maintained in the range of 3.0–3.5 (data not shown). The enzymes were reacted with H_2_O_2_/guaiacol and then subjected to LC-MS/MS analysis. Forty-six percent of the protein sequence was found to be covered through the mass analysis. Trypsin-digested WT samples (control and inactivated ones) subjected to Q-TOF MS showed several peptide ions (details about peptide fingerprinting are shown in Additional file 1: Figure S1a, b). Peptides of *m/z* 566.9209 and 589.5857 from WT-control and WT-inactivated, respectively, both were sequenced as **TACEWQSFVNNQSK** (Table 1). Compared with WT-control, y10 ion of the WT-inactivated sample showed a mass shift of +125 Da due to 2 deamidated sites (Q252 and N256) and 1 moiety guaiacol radical coupling with W251 site.

**Table 1:** Liquid chromatography-tandem mass spectrometry analysis of peptide from control and inactivated samples

Samples	Observed peak (*m/z*)	Molecular weight (Da)		Sequence and modification
		Expected	Calculated	
WT-control	566.9209	1694.7409	1697.7468	TACEWQSFVNNQSK C3: Carbamidomethyl
WT-inactivated	589.5857	1765.7353	1765.7379	TACEWQSFVNNQSK W5: 1Guaiacol, Q6: Deamidated, N10: Deamidated
W251A-inactivated	571.2548	1710.7426	1709.6852	TACEAQSFVNNQSK F8: 1Guaiacol
A242D-inactivated	487.7306	1946.8934	1947.772	TACEWQSFVNNQSK W5: 1Guaiacol, F8: 1Guaiacol

### The role of W251 in intramolecular electron transfer

**Fig. 1:**
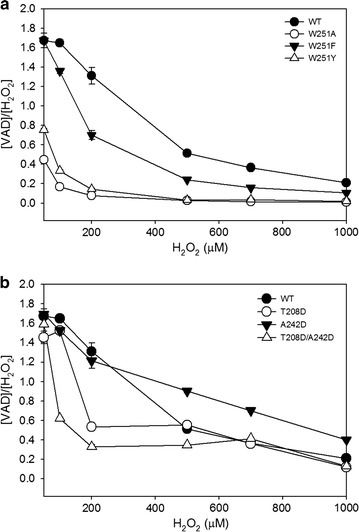
H_2_O_2_-dependent oxidation of VE dimer catalyzed by WT, the mutated W251 variants (**a**) and the mutated variants surrounding W251 (**b**). Oxidation of VE dimer (2000 µM) was catalyzed by 0.075 µM enzyme in the presence of various H_2_O_2_ concentrations. The reaction was performed in 0.1 M tartrate buffer at pH 4.0 and subjected to HPLC analysis for detection of formed VAD after 4 h. The theoretical stoichiometric ratio is described as 2:1 for [VAD]:[H_2_O_2_]

Coupling between the W251 site and guaiacol was found only in inactivated sample, which implies that W251 turns into a radical intermediate during the catalysis cycle of LiPH8. Here, role as electron station for hopping ET has been approved again when W251 was mutated into aromatic amino acids such as Phe or Tyr which relatively retained the steady-state kinetics of the oxidation of VE dimer (Table 2). However, comparing with wild-type, mutant W251F and W251Y showed lower efficiency in conversion yield of VE dimer at high concentration of H_2_O_2_ (Fig. 1a).

**Table 2:** Steady-state kinetic parameters for the oxidation of VE dimer for wild-type and mutants

Variants	Oxidation of VE dimer		
	K_M_ (mM)	k_cat_ (s^−1^)	k_cat_/K_M_ (s^−1^ mM^−1^)
WT	0.13 ± 0.03	0.77 ± 0.05	5.59 ± 0.69
W251A	0.26 ± 0.01	0.06 ± 0.01	0.25 ± 0.04
W251F	0.15 ± 0.01	0.61 ± 0.08	4.10 ± 0.52
W251Y	0.16 ± 0.01	0.45 ± 0.01	2.81 ± 0.05
T208D	0.38 ± 0.01	2.44 ± 0.08	6.40 ± 0.11
A242D	0.55 ± 0.02	16.48 ± 0.20	29.96 ± 0.92
T208D/A242D	1.22 ± 0.03	16.13 ± 0.35	13.22 ± 0.31

Furthermore, tenfold lower k_cat_ value in the oxidation of VE dimer was observed for mutant W251A when compared to wild-type (Table 2). The mutation of W251 into Ala also caused a change in the occurrence of the intramolecular electron transfer, which was characterized by the spontaneous decay rate constant of compound I in the transient-state dropping from 3.854 s^**−1**^ to 0.583 s^**−1**^ (Table 3). It was also confirmed that W251A-containing peptide did not show coupling with guaiacol when oxidation and LC-MS/MS analysis were performed in the same condition (Table 1 and details about peptide fingerprinting are shown in Additional file 1: Figure S1c).

**Table 3:** Transient-state kinetic constants for the reduction of compound I by H_2_O_2_ for wild-type and mutants

Mutants	k_obs_ (s^−1^)
WT	3.854 ± 0.188
W251A	0.583 ± 0.019
A242D	4.125 ± 0.203

Although the possibility that sites other than W251 may form radical–radical coupling cannot be excluded because peptide coverage was only 46%. However, it can be concluded that post-catalysis modification with guaiacol radical only involves in the aromatic character of W251 site.

**Fig. 2:**
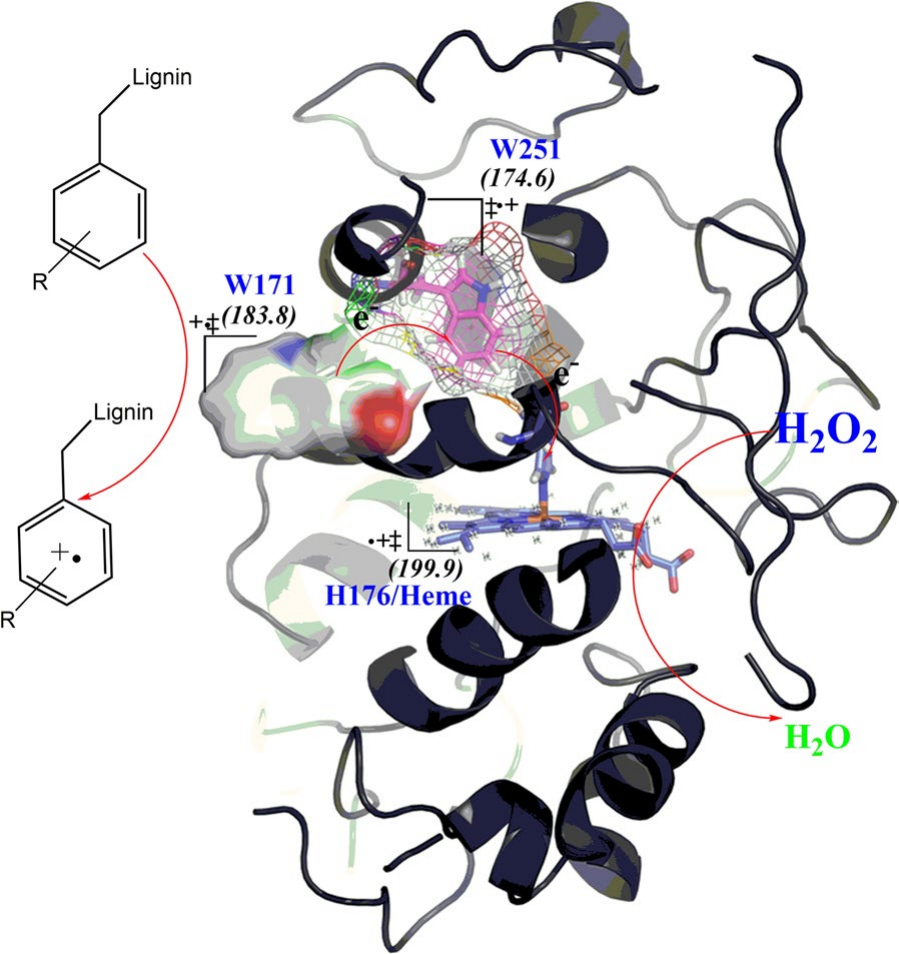
Proposed electron transfer pathway through the electron-relay W251 in catalysis of lignin substrate. *Bold*, *italic indexes* in parenthesis which indicated the energy differences for one-electron transfer reaction at each redox centers were calculated in the gas phase (∆G^0^, Kcal mol^−1^)

As formation of radical intermediate during catalytic cycle, W251 was proposed as one electron-relay of the one-electron transfer pathway between H176/Heme and W171 (Fig. 2). The barrier energies (∆G^0^) calculated for the critical redox centers (H176/Heme, W171, and W251) approved W251 as an energetically favorable electron-relay in the LRET (Fig. 2).

### Facilitating acidic environment around the W251 site

**Fig. 3:**
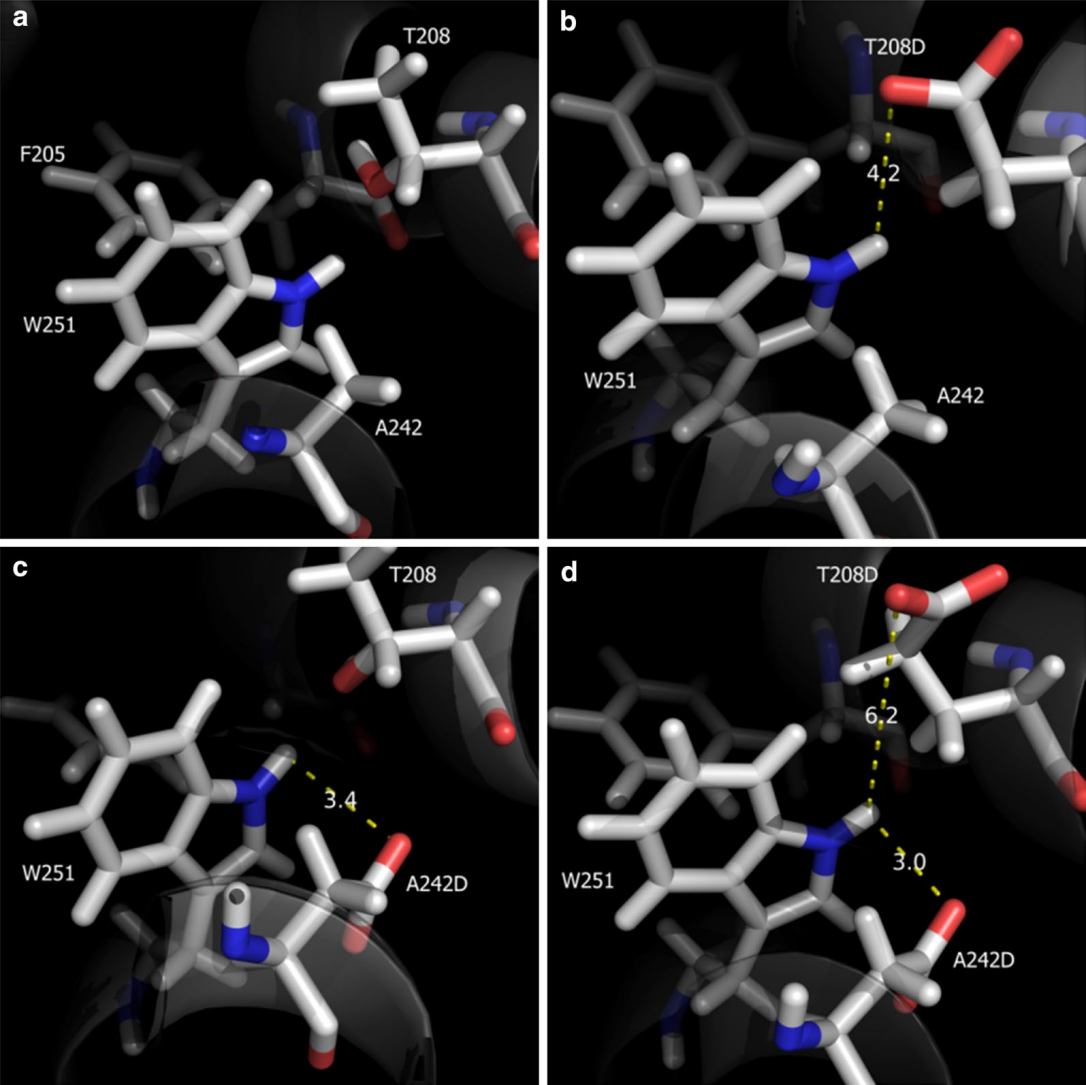
Refined modeled structure of wild-type (**a**), as well as the mutants T208D (**b**), A242D (**c**), and double mutant T208D/A242D side-chain structures (**d**), were visualized as CPK-colored sticks by Molegro molecular viewer software

Installation of an acidic microenvironment around W251 resulted in a significant difference in the catalytic efficiency for the oxidation of the VE dimer (Table 2). The model structures of mutants suggested the rational mutations of T208 and/or A242 into Asp residues which exhibited the closed interactions with W251 (Fig. 3). Improvement of the k_cat_ value was observed in the A242D mutant for the oxidation of the VE dimer. Mutant A242D, among many mutants, showed exceptional catalytic performance by yielding 21.1- and 4.9-fold higher increases in k_cat_ and k_cat_/K_M_ values, respectively, in the oxidation of the model lignin dimer. Furthermore, comparing with WT LiPH8, mutant A242D could retain rather higher efficiency in the oxidation of VE under the excess H_2_O_2_ (Fig. 1b). However, an increased acidity contribution by the double mutant T208D/A242D did not show a synergistic increase in the oxidation of the VE dimer (Fig. 1b).

Although exhibiting higher activity, the mutant A242D still showed the covalent bonding with guaiacol radical at site W251, which was confirmed by the LC-MS/MS analysis at the similar condition (Table 1 and details about peptide fingerprinting are shown in Additional file 1: Figure S1d).

## Discussion

### W251 residue: accelerating the intramolecular electron transfer and being intrinsically radical susceptible

The coupling occurrence between W251 and guaiacol was detected only in the inactivated sample (addition of H_2_O_2_) and only with aromatic residue, which confirmed that the W251 radical was formed during the catalysis cycle of LiPH8. The combination of rational mutations (W251F, W251F, and W251A), steady-state/transient kinetics, and the computationally calculated energies for formation of cationic radical demonstrated that W251 plays a key role as a stepping stone in the electron transfer route between W171 and heme by following a hopping ET mechanism (Fig. 2).

**Fig. 4:**
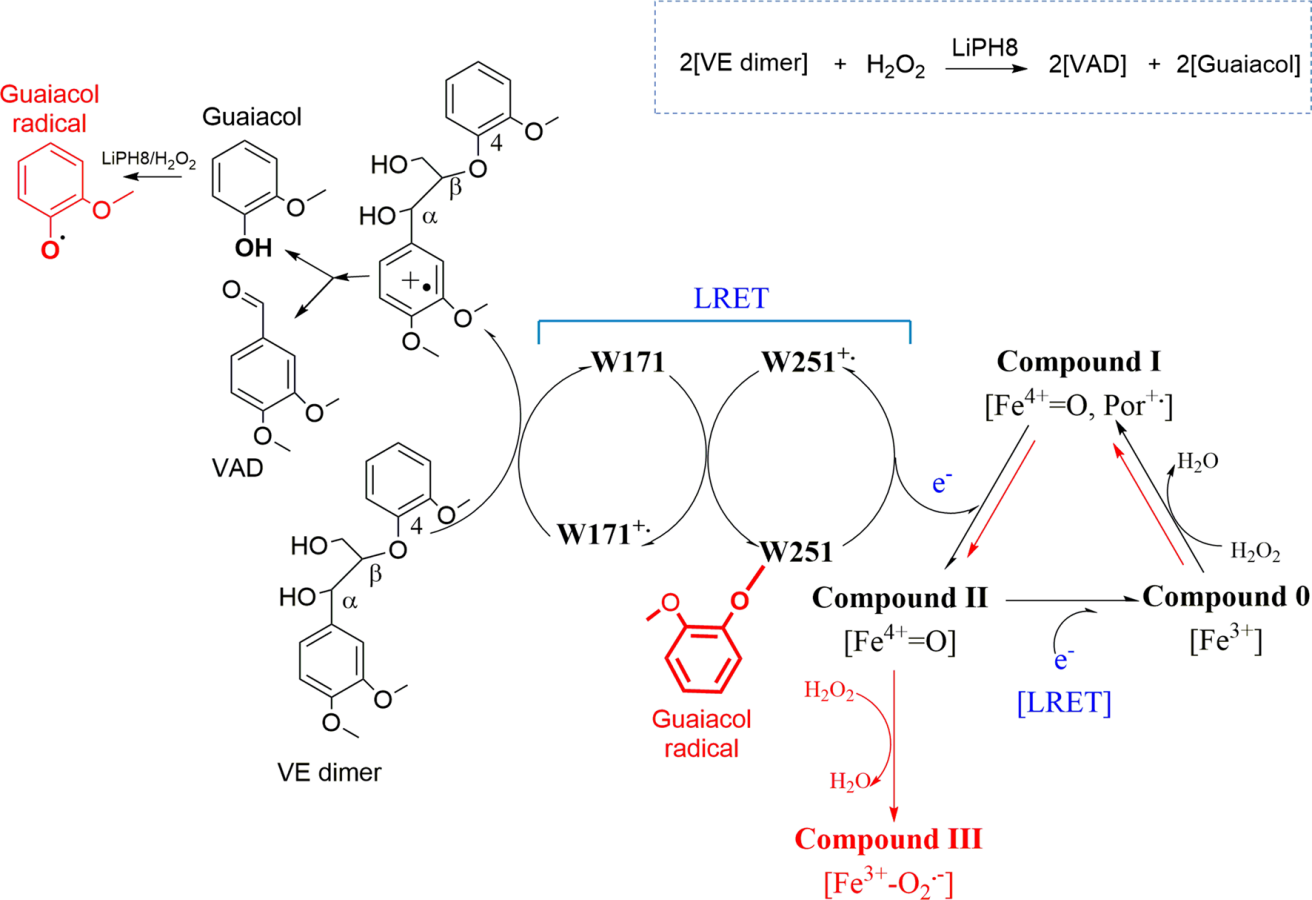
Closed catalysis cycle and the inhibiting mechanism by guaiacol in LiPH8-catalyzed degradation of VE dimer. Under catalysis of LiPH8/H_2_O_2_, VAD and guaiacol were detected as released products from degradation of VE dimer. The paths depicted in red lead to the formation of inactive compound III when suicide coupling between W251 and guaiacol occurs. In the closed catalytic cycle, the stoichiometric ratio is described as 1:2:2 for [H_2_O_2_]:[VAD]:[Guaiacol]

During catalytic cycle, LiPH8 harbors W251 radical which helps for a facile LRET between surface-active site W171 and Heme. However, this susceptible redox center can also be attacked by oxidative species during oxidation reaction. The β-*O*-4 bond cleavage of VE dimer released guaiacol and the inert chemical, VAD. The unexpectedly subsequent oxidation of guaiacol generated the guaiacol radical which covalently bonded with W251. The suicide modification of W251 by guaiacol radical resulted in the loss of its electron-relay property. Then, the oxidation of high-redox potential substrate such as VE dimer was suppressed and the presence of excess H_2_O_2_ concentration led to a formation of inactive compound III rather than a closed catalysis cycle (paths depicted as red in Fig. 4).

The suicide modification during catalysis cycle has been reported for oxidoreductases which harbor susceptible amino acids including methionine, cysteine, tryptophan, phenylalanine, tyrosine, and histidine [17]. A concrete evidence for suicide coupling between enzymes and phenoxy radicals was recently described for horseradish peroxidase C and fungal peroxidase from *Coprinus cinereus*. Horseradish peroxidase C catalyzes a lignin polymerization reaction at neutral pH conditions, which is more favorable for the generation/coupling reaction of phenoxy radicals [18]. Interestingly, a self-destructive coupling between LiPH8 and phenoxy radical at low pH 4.0 was firstly reported in this study. This novelty revealed inhibiting mechanism helps to coordinate mechanism-based protein engineering work for an efficient degradation of lignin.

The electron-relay can render the distant ET a multistep tunneling process in which the kinetics are faster in comparison to one long single-step electron transfer between the donor and the acceptor. Without the presence of aromatic amino acids such as Phe or Tyr or Trp, the gap between HOMO and LUMO levels do not appear to facile a transport of electrons [19]. For example, the oxidation of Cu^I^ by electronically excited Re^I^ is 100-fold faster than single-step ET due to the transient oxidation of W122, which was confirmed in case of azurin protein from *Pseudomonas aeruginosa* [20].

Deprotonation-coupled ET leads to the formation of neutral radical rather than cation radical, which is favorable for covalent coupling with phenoxy radical. Compared with Phe and Tyr, Trp shows higher tendency to produce Trp^+^ in aqueous solution through one-electron ET process [21]. This explained why W251F and W251Y still rendered ET process but exhibited lower oxidation efficiency due to more possibilities in coupling with guaiacol radical (Fig. 1a).

### Manipulating microenvironment of electron-relay for a facile electron transfer

The radical cations thus produced are only stable up to a few hundred nanoseconds and chiefly decay by deprotonation, yielding phenoxyl radicals. The reaction solvent and its microenvironment directly affect the stability and reactivity of the corresponding radical cations [22]. The polarizability, resonance, and charge density are factors that can stabilize radical cations. The surface-active site W171 of LiPH8 was a good demonstration, where its acidic microenvironment was prepared by E168, E250, and D264. This created a unique physicochemical property of a cationic radical and high-redox potential intermediate in W171 [3]. Unexpectedly unlike W171, more local acidic groups in double mutant T208D/A242D did not show a proportional increase in the oxidation of the VE dimer. We supposed that in the double mutant T208D/A242D, the titratable groups at these sites are strongly coupled (Fig. 3d). This may cause unfavorable energy because either both of them are protonated or deprotonated, which was proved in the Monte Carlo titration calculation [23].

**Fig. 5:**
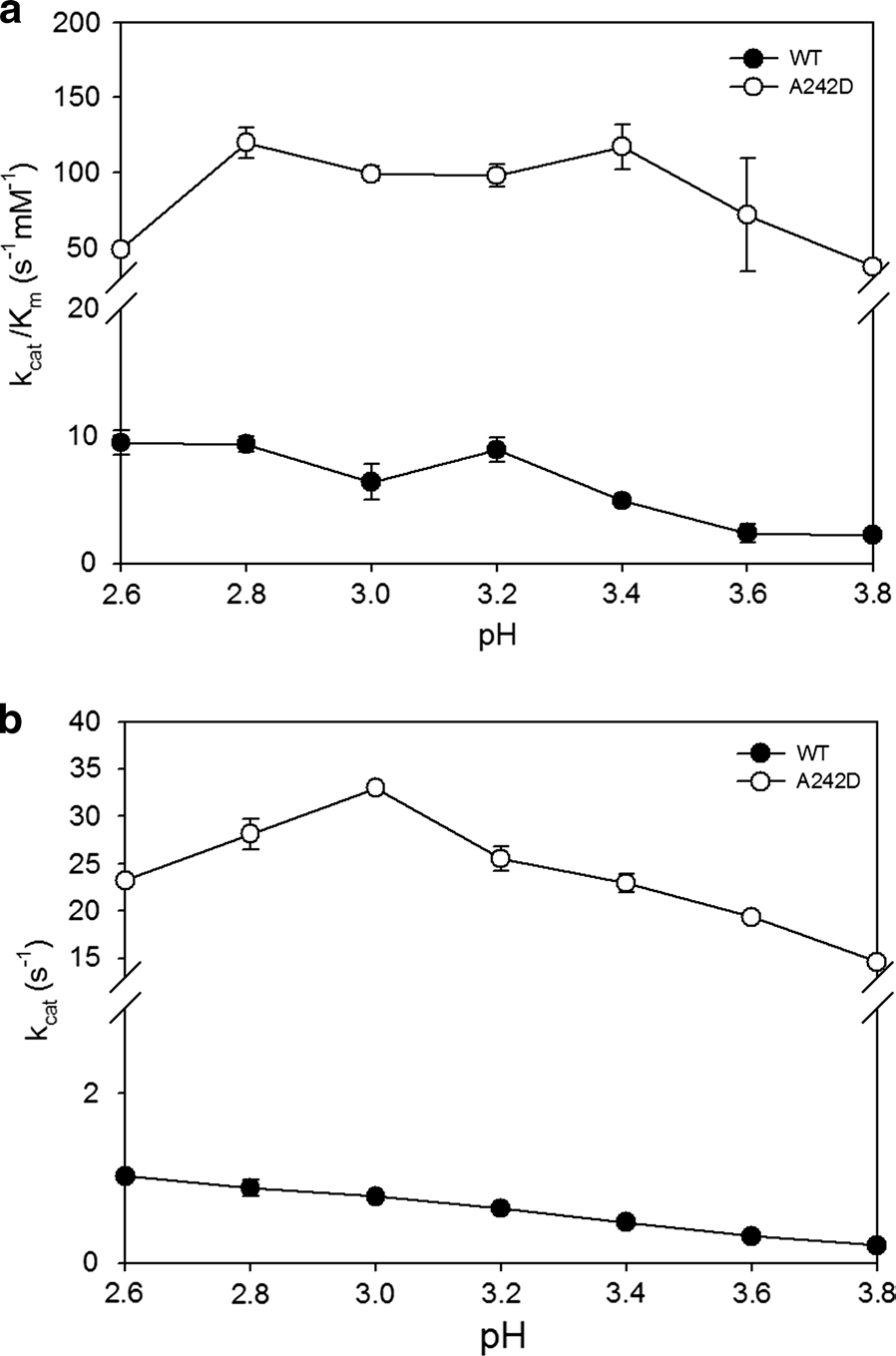
pH-dependent steady-state kinetic parameters for wild-type and the A242D mutant. The enzyme activity was presented as k_cat_/K_M_ (**a**) and k_cat_ (**b**) values for oxidation of VE dimer

To understand the role of the A242D site in LiPH8, pH-dependent oxidations of VE dimer were investigated. The wild-type and mutant A242D shared the similar profile of catalytic efficiency with VE dimer (Fig. 5a). However, only A242D exhibited bell-shaped patterns in the pH-dependent turnover values (Fig. 5b). The bell-shaped profile of k_cat_ variation with pH in mutant A242D reflects the alteration of the ionizable state of A242D site in active site W251 which participated in catalysis of VE dimer. It is demonstrated that pH-dependent conformation of A242D site concerted in hydrogen bonding with W251, which may keep W251 at a right position for optimal energy geometry in the occurrence of intramolecular ET.

Apparently, due to being buried in the protein interior, the titrated state of the A242D site depends on the dominant factor from its surrounding titratable groups. The pK_a_ value of A242D was empirically predicted from applying an environmental perturbation (ΔpK_a_) to the unperturbed intrinsic value of the group (pK_model_) according to the following equation, where ΔpK_a_ value was calculated from the combined effects of desolvation, hydrogen bonding, and charge–charge interaction:

$$pK_{a} = pK_{\bmod el} + \Delta pK_{a}.$$

**Fig. 6:**
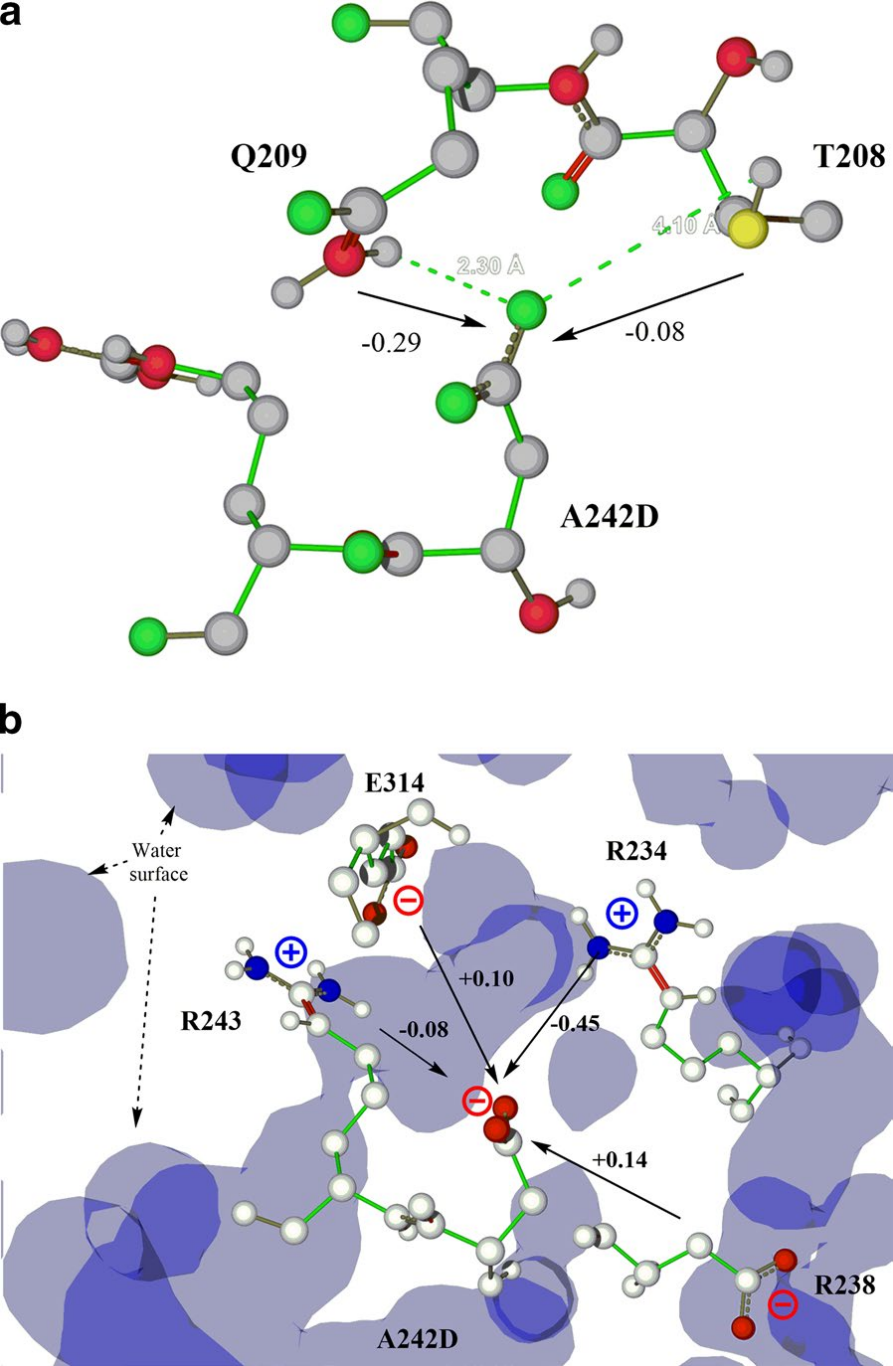
Interaction between A242D and its surrounding residues: **a** hydrogen bonding and **b** charge–charge interaction. *Numbers aligned with arrows* indicate the pK_a_ shift effect on A242D

Herein, the pK_a_ shift effects by surrounding residues such as T208, Q209 (hydrogen bonding), R234, D238, R243, and E314 (charge–charge interaction) were investigated (Table 4). Additional studies of the effects of these ionizable groups, especially the exposed site R243 and partially buried Q314, on the titrated state of A242D should be conducted to engineer the redox-active state of the electron-relay W251 (Fig. 6).

**Table 4:** Predicted pK_a_ value of the A242D site and specific ΔpK_a_ terms of its surrounding residues

Site	pK_a_	pK_model_	Desolvation effect		Hydrogen bonding		Charge–charge interaction
			Global	Local	Side chain	Backbone	
A242D	8.83	3.8	4.36	1.33	T208 (−0.08) Q209 (−0.29)		N234 (−0.45) D238 (+0.14) N243 (−0.08) E314 (+0.10)

Values in brackets indicate the pK_a_ shift effect of each residue

### Suggestion of multiply bridged electron transfer pathway

**Fig. 7:**
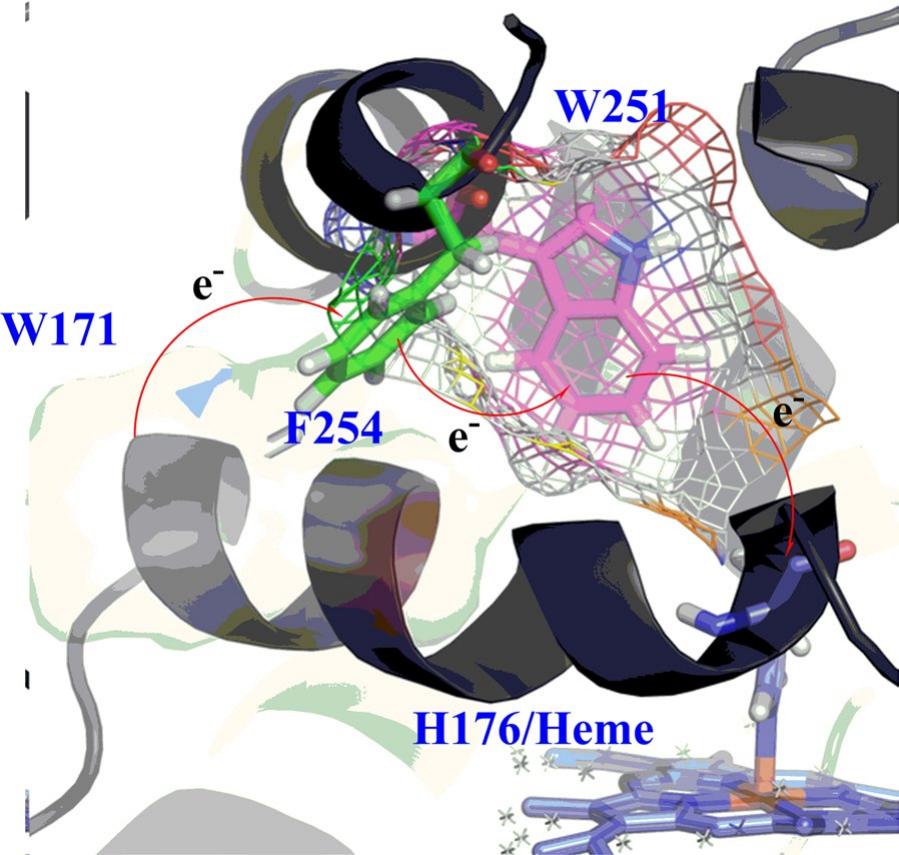
Proposed multistep tunneling process in LRET between W171 and Heme through W251 and F254

Besides W251, the radical coupling between F254 and guaiacol was found in mutants W251A and A242D but not found in WT (Table 1). Mutations W251A and A242D may cause an alteration in structural conformation and redox properties of other local residues. In this context, F254 was suggested as another ET relay on the LRET which was manipulated through the mechanism of multi-redox center tunneling process. Further study on the construction of an optimized and radical-robust ET tunneling process should be conducted for higher efficiency in degradation of lignin (Fig. 7).

## Conclusion

Using combination of liquid chromatography-tandem mass spectrometry, rational mutagenesis and characterization of transient/steady-state kinetic parameters demonstrate that (i) the covalent bonding between the released product and the intramolecular W251 electron-relay caused suicide inhibition mode during degradation reaction of non-phenolic lignin dimer and (ii) manipulating the acidic microenvironment around radical-damage active site successfully improves catalytic efficiency in oxidation of non-phenolic lignin dimer. The results obtained demonstrate interesting and potential approach of engineering lignin peroxidases to protect active sites which are easily attacked by the released radical product. Radical-robust mutants exhibit potentialities in industrial utilization for delignification of not only lignin model dimer but also real lignin structure from biomass waste sources.

## Additional file

**Additional file 1: Figure S1.** Q-TOF MS analysis of Trypsin-digested lignin peroxidase samples (350–1200 *m/z*). The details about peptide fingerprinting for WT_control, WT_inactivated, mutant W251A and mutant A242D shown in Fig S1a, b, c and d, respectively.

## Abbreviations

LiP: lignin peroxidase
VP: versatile peroxidase
VE dimer: veratrylglycerol-beta-guaiacyl ether
VA: veratryl alcohol
LRET: long-range electron transfer
ABTS: 2,2-azino-bis (3-ethylbenzothiazoline-6-sulfonate
LC-MS/MS: liquid chromatography-tandem mass spectrometry
CBB: Coomassie brilliant blue G-250
VAD: veratraldehyde
IEF_PCM: integral equation formalism polarizable continuum model
DFT: density functional theory
