# Supplementary material for: Improvement of catalytic performance of lignin peroxidase for the enhanced degradation of lignocellulose biomass based on the imbedded electron-relay in long-range electron transfer route
Source: Biotechnol Biofuels. 2016 Nov 15;9:247. doi: 10.1186/s13068-016-0664-1 (PMC5111271; doi:10.1186/s13068-016-0664-1)
Supplement: Supplementary file 1 — Additional file 1: Figure S1. Q-TOF MS analysis of Trypsin-digested lignin peroxidase samples (350–1200 m/z). The details about peptide fingerprinting for WT_control, WT_inactivated, mutant W251A and mutant A242D shown in Fig S1a, b, c and d, respectively. [file 13068_2016_664_MOESM1_ESM.docx]

**SUPPLEMENTARY INFORMATION**

**
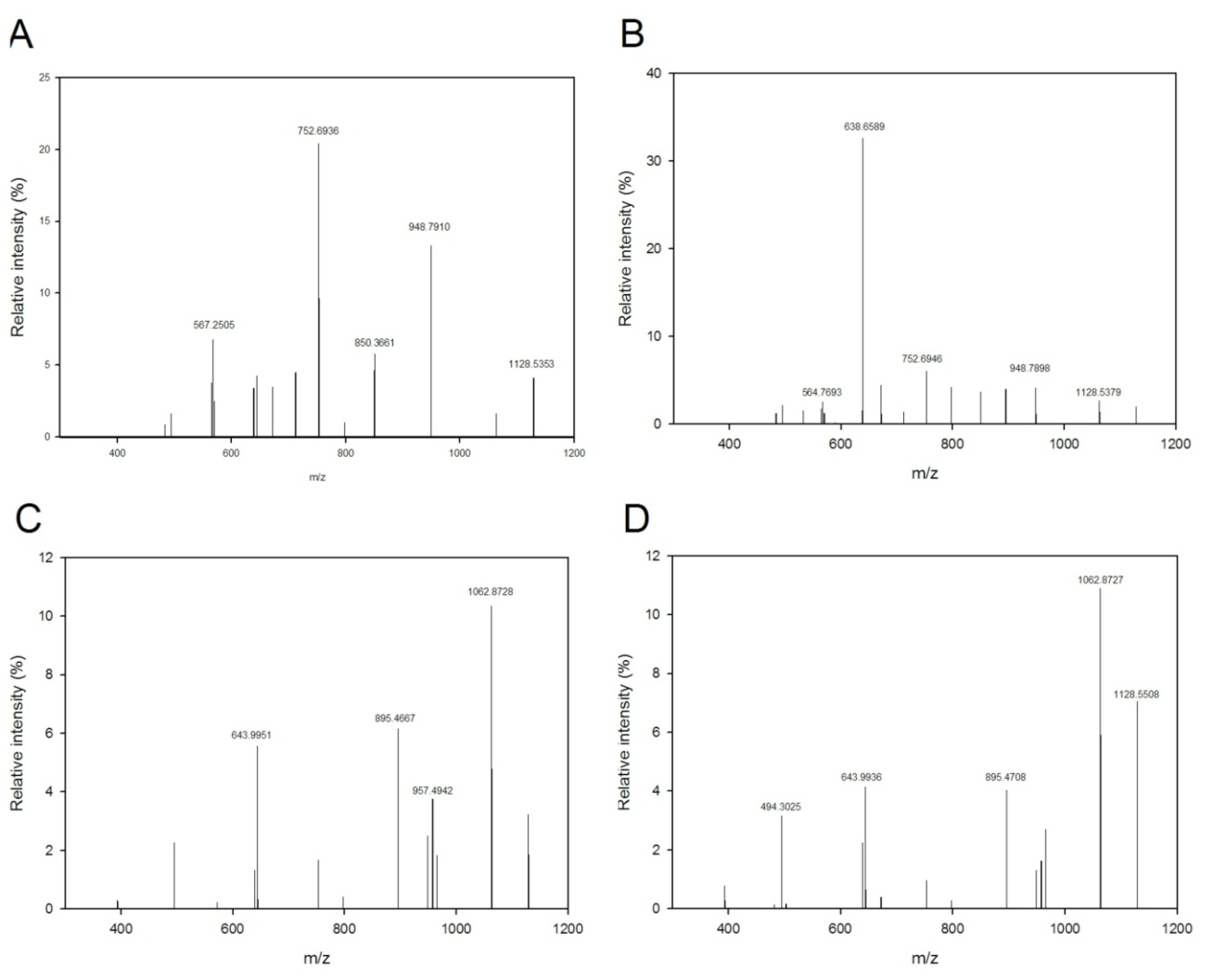
**

**Figure S1. Q-TOF MS analysis of Trypsin-digested lignin peroxidase samples (350 – 1200 m/z).** The details about peptide fingerprinting for WT_control, WT_inactivated, mutant W251A and mutant A242D shown in Figure S1_A, S1_B, S1_C and S1_D, respectively.
